# Successful synthesis of a glial‐specific blood–brain barrier shuttle peptide following a fragment condensation approach on a solid‐phase resin

**DOI:** 10.1002/psc.3448

**Published:** 2022-08-28

**Authors:** Othman Al Musaimi, Sophie V. Morse, Lucia Lombardi, Simona Serban, Alessandra Basso, Daryl R. Williams

**Affiliations:** ^1^ Department of Chemical Engineering Imperial College London London UK; ^2^ The Sargent Centre for Process Systems Engineering Imperial College London London UK; ^3^ Department of Bioengineering Imperial College London London UK; ^4^ Purolite Ltd Llantrisant UK

**Keywords:** blood–brain barrier shuttle, cell‐penetrating peptides, fragment condensation, solid‐phase peptide synthesis

## Abstract

Successful manual synthesis of the TD2.2 peptide acting as a blood–brain barrier shuttle was achieved. TD2.2 was successfully synthesised by sequential condensation of four protected peptide fragments on solid‐phase settings, after several unsuccessful attempts using the stepwise approach. These fragments were chosen to minimise the number of demanding amino acids (in terms of coupling, Fmoc removal) in each fragment that are expected to hamper the overall synthetic process. Thus, the hydrophobic amino acids as well as Arg(Pbf) were strategically spread over multiple fragments rather than having them congested in one fragment. This study shows how a peptide that shows big challenges in the synthesis using the common stepwise elongation methodology can be synthesised with an acceptable purity. It also emphasises that choosing the right fragment with certain amino acid constituents is key for a successful synthesis. It is worth highlighting that lower amounts of reagents were required to synthesise the final peptide with an identical purity to that obtained by the automatic synthesiser.

## Graphical abstract

Successful manual synthesis of the TD2.2 peptide, which acts as a blood–brain barrier shuttle, was achieved by sequential condensation of four protected peptide fragments on a solid‐phase resin. The chosen fragments minimised the number of demanding amino acids in each fragment, facilitating the overall synthetic process.

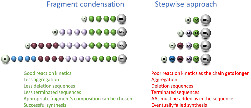

## 1 INTRODUCTION

The advent of cell‐penetrating peptides (CPPs), also called protein transduction domains (TDs), has allowed the transfer of various cargos into cells, ranging from small molecules to large proteins.^1^ This strategy has helped deliver inherently hydrophilic molecules that cannot cross hydrophobic barriers—as the brain one—by themselves. Cargos can be conjugated to the CPP either covalently, which is based on chemical bond formation (amide, disulfide, thioester, etc), or non‐covalently, which is based on electrostatic and/or hydrophobic interactions between oppositely charged molecules (the CPP and the cargos).^2^ Each approach has its pros and cons;^1^ thus, choosing the appropriate approach is mainly dependent on the type of cargo to be delivered and the extent to which its biological activity can be influenced by the conjugating strategy.^2^

The capability to cross complex physiological barriers and transport cargo can be controlled by changing the length of the peptide sequence and/or charge.^3^ CPPs are usually short amphipathic sequences containing both hydrophobic and positively charged amino acids. A desirable CPP passes through biological barriers in a noninvasive manner without cell rupture.^4^ The internalisation mechanism is not fully understood and various studies showed more than one mechanism is involved.^5^, ^6^ The two main routes of CPP uptake are passive translocation and endocytosis. Studies successfully demonstrated the application of these sequences in various fields including imaging agents, diagnosis, delivery of siRNA, anticancer drug, nanoparticles, antimicrobial and antifungal action.^1^, ^7^, ^8^, ^9^, ^10^

The literature also reports numerous examples of CPPs used to transport drugs to the brain. In order to reach the brain, peptides must overcome a major obstacle: the blood–brain barrier (BBB). The BBB is an important structure in the brain that maintains homeostasis, protecting the brain parenchyma from toxic compounds.^11^ However, it also restricts drugs and imaging agents from reaching their therapeutic targets,^11^, ^12^ especially when their molecular weight is above 400 Da.^13^, ^14^ To overcome this dilemma, various approaches have been developed, with different success rates in terms of the efficiency of the delivery process itself and its accompanied safety consequences.^12^ BBB shuttle peptides (BBBSp) are a special class of CPPs and are considered a promising tool to deliver therapeutic agents noninvasively through the BBB with a tolerable safety profile and without affecting or compromising the integrity of the BBB.^4^, ^12^, ^15^ They are able to cross the BBB as well as the cellular membrane of the therapeutic target.^12^, ^16^

Peptides are gaining a prime position in the pharmaceutical industry with a total of 22 peptides approved by the US Food and Drug Administration (USFDA) in the last 6 years.^17^ This is ascribed to the continuous development and advancements in synthesis methods, known as solid‐phase peptide synthesis (SPPS), the method of choice for preparing peptides at research and industrial scales.^18^ Despite the easiness that has been witnessed as a result of introducing the automatic synthesiser,^19^ there is sometimes a need for manual synthesis, especially when preparing short peptides with modifications that cannot be done with the automatic synthesiser. Furthermore, unlike the automatic approach, by using manual synthesis, green solvents and reagents can be incorporated easily and the protocol is optimised as needed.^20^, ^21^, ^22^, ^23^

In SPPS strategy, peptides are assembled on a solid support in a stepwise manner where repetitive cycles of coupling, deprotection and washing steps are carried out.^24^ Although the stepwise approach has been proven effective, its efficiency often deteriorates as the chain gets longer, beyond the 30 amino acids and sometimes beyond 20 depending on the amino acid constituents. This is due to the structurally related deletion, termination and modification sequences that are generated at each synthetic step, which could eventually drive the whole process to failure.^25^

The convergent approach is considered an alternative and more favourable approach to deliver pure peptides in the case of long peptides (20 amino acids and more), which are difficult to obtain following the common stepwise strategy.^26^ In some cases, the convergent approach avoids undesired side reactions to circumvent such drawbacks. For example, in an effort to green the SPPS methodology in Albericio's group,^27^ they observed an undesired acylation of less hindered Gly residue when using green γ‐valerolactone (GVL) solvent instead of *N*,*N*‐dimethylformamide (DMF). Thus, to avoid this problem, they introduced Gly‐containing dipeptide (Gly at the C‐terminus) rather than Gly on its own. They were able to prepare a pure demanding ABRF1992 peptide, which comprises five Gly residues in its sequence. The same group has also utilised the fragment condensation approach to prepare a liraglutide peptide with better purity suitable for production scale. It is worth highlighting their elegant approach to protect the C‐terminus of the peptide, where they utilised a multidetachable linker for this purpose.^28^ Gatos et al. considered the convergent approach to deliver pure Tyr^0^‐Atriopeptin II peptide by assembling peptide fragments with no β‐turn in their sequence. Due to this approach, they were able to synthesise higher yield and purity of the target peptides by avoiding various difficulties ascribed to the presence of β‐turn regions, such as serious coupling difficulties and inefficient Fmoc‐removal.^29^ As semaglutide contains an unnatural amino acid which hinders the fermentation process, SPPS is the main route for its synthesis.^30^ Its synthesis, however, has several shortcomings such as low yield and the presence of several deletion sequences which complicate the purification process and imply additional costs penalty on the overall process. Therefore, fragment condensation in solution was successfully exploited to deliver Aib^8^‐Arg^34^‐GLP‐1 (7–37) with better yield and purity than the common SPPS.^31^

**FIGURE 1:**
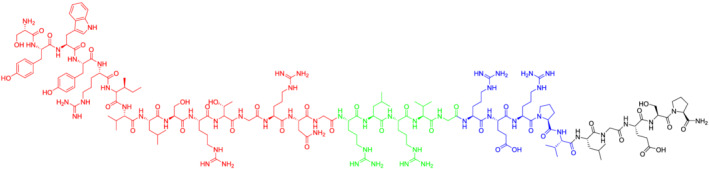
Chemical structure of TD2.2 BBBSp (H‐SYWYRIVLSRTGRNGRLRVGRERPVLGESP‐NH_2_). Four fragments to be condensed: red: SYWYRIVLSRTGRNG (15 residues) on CTC, green: RLRVG (5 residues) on CTC, blue: RERPV (5 residues) on CTC, black LGESP (5 residues) on Rink amide resin

TD2.2 has been reported to target oligodendrocytes and has been shown to not target nonglial cells, such as human neural cells and human dermal fibroblasts (Figure 1).^32^ This peptide has therefore high specificity compared with other CPP or BBBSp.^33^ The usefulness of this BBBSp has been previously proven in vitro^32^ (the peptide was expressed in *E. coli* cells), and recently, it is being investigated in vivo by the authors of this manuscript (data not shown).

Here, we report the first chemical synthesis of the TD2.2 BBBSp via SPPS. We demonstrated the effectiveness of the fragment condensation approach versus the stepwise approach and the benefits over the automatic approach. The synthesis was done manually following the fragment condensation approach on solid‐phase settings. For instance, while the stepwise approach failed to render the target peptide, identical purities were obtained with the fragment condensation approach and when using the automatic synthesiser.

## 2 MATERIALS AND METHODS

PuroSynth CTC (1.0 mmol/g, supplier's specification) and PuroSynth Rink Amide (0.5 mmol/g, supplier's specification) resins were used for all syntheses. Automatic peptide synthesis was carried out using Liberity Blue™ automated microwave‐assisted peptide synthesiser (CEM). All reagents and solvents were obtained from commercial suppliers and were used without further purification unless otherwise stated. Analytical HPLC was performed on Shimadzu LC20 system using Lab solution software for data processing. Column: Symmetry Luna C18 (3.6 μm, 4.6 × 150 mm) column, with flow rate of 1.0 ml/min and UV detection at 220 nm. Mobile phase A was 0.1% trifluoroacetic acid (TFA) in H_2_O, and mobile phase B was 0.1% TFA in CH_3_CN. LCMS of the final peptide was performed on Waters Synapt G2 mass spectrometer using a nanoEase HSS C18 (1.8 μm, 75 μm × 150 mm) column, and data processing was carried out by MassLynx software. Buffer A was 0.1% formic acid in H_2_O, and buffer B was 0.1% formic acid in CH_3_CN. The mass of the peptide fragments was performed on Velos Pro (ThermoFisher Scientific), a hybrid linear trap quadrupole (LTQ)‐Orbitrapmass spectrometer. The samples were directly infused into the system.

### 2.1 Incorporation procedure

#### 2.1.1 CTC resin

First, amino acids were incorporated onto CTC resin using dry CH_2_Cl_2_. CTC resin was swelled in CH_2_Cl_2_ for 10–20 min. The Fmoc‐amino acids (2 equiv) were dissolved in a minimum amount of the CH_2_Cl_2_ (0.5 ml/100 mg resin) and sonicated for 10 min. Four equiv. of *N*,*N*‐diisopropylethylamine (DIEA) was then added to the solution, which in turn was added to the previously swelled resin and allowed to react for 1 h under mechanical shaking. After this, MeOH (80 μl/100 mg of resin) was added to endcap any unreacted chloride of the CTC resin. Finally, the resin was washed twice with CH_2_Cl_2_ and dried over vacuum.

#### 2.1.2 Rink amide resin

First, amino acids were incorporated onto Rink amide resin using dry DMF. Rink amide resin was swelled in DMF for 10–20 min. The Fmoc was removed using 20% piperidine/DMF and the mixture was allowed to shake for 2 and 7 min. The Fmoc‐amino acids (3 equiv) and OxymaPure (3 equiv) were dissolved in a minimum amount of the DMF (0.5 ml/100 mg resin) and sonicated for 10 min. Three equiv. of *N*,*N*‐diisocarbodiimide (DIC) was then added to the solution, which in turn was added to the previously swelled resin and allowed to react for 1 h under mechanical shaking. Finally, the resin was washed twice with CH_2_Cl_2_ and dried over vacuum.

#### 2.1.3 Peptide synthesis

Peptides were synthesised following the standard methodology performed in our laboratory (3 equiv. of Fmoc‐AA‐OH, 3 equiv. of OxymaPure, 3 equiv. of DIC) or (2 equiv. of Fmoc‐AA‐OH, 1.9 equiv. of (1‐cyano‐2‐ethoxy‐2‐oxoethylidenaminooxy)dimethylamino‐morpholino‐carbenium hexafluorophosphate (COMU), 4 equiv. of DIEA) in DMF, and then shaking for 1 h. Fmoc was then removed as per Section 2.1.2. All Arg and the residue that comes after were double coupled to ensure complete coupling.

#### 2.1.4 Fragment condensation

Fragments were condensed using 2 equiv. of OxymaPure, 2 equiv. of DIC with respect to the Rink amide resin loading, in DMF, and then the mixture was allowed to shake for 24 h. To ensure the completion of the coupling, fresh quantity of the DIC was added to the reaction mixture after the first 12 h. Fmoc was removed as per Section 2.1.2; however, for 30 twice.

#### 2.1.5 Automatic synthesis

Coupling of the amino acid, to the growing peptide chain, was achieved through addition and heating of the Fmoc‐AA‐OH acid (0.25 mmol, 5 equiv., 0.2 M in DMF), (OxymaPure, 0.25 mmol, 5 equiv., 0.5 M in DMF), and DIC (0.50 mmol, 10 equiv., 0.5 M in DMF) at 90°C for 2 min (single coupling) or 2 × 2 min (double coupling). N‐terminal deprotection of the growing peptide chains was achieved through Fmoc‐cleavage via addition of piperidine (20% v/v in DMF) and in OxymaPure (0.1 M in DMF) and heating at 90°C for 1.5 min.

### 2.2 Cleavage protocols

#### 2.2.1 Protected fragments

Peptide resin was placed in a syringe fitted with porous polyethylene disc. It was then swelled with CH_2_Cl_2_ for 10 min. The solvent was then filtered off, the cleavage solution (2 ml, 2% TFA in CH_2_Cl_2_) was added per 100 mg of the peptide resin and the syringe was closed with a cap and shaken for 30 min at rt. Finally, the filtrate was collected over water (10.0 ml) and was lyophilised for the subsequent fragment condensation step.

#### 2.2.2 Unprotected fragments

The final synthesised peptide was cleaved from the resin using TFA/triisopropylsilane (TIS)/H_2_O (95:2.5:2.5) (1 ml/100 mg) under mechanical shaking for 1 h. Chilled diethyl ether was then added (five times the cleavage solution volume), and the solution was kept in an ice bath for 30 min. The solution was then centrifuged for 5 min at 5000 rpm, and the supernatant was decanted. A new amount of the ether (five times the cleavage solution volume) was added to repeat this step. Any remaining ether was dried under N_2_. Finally, the precipitate was dissolved in water. A small amount of the solution was injected into HPLC system to check the purity of the final product.

## 3 RESULTS AND DISCUSSION

**FIGURE 2:**
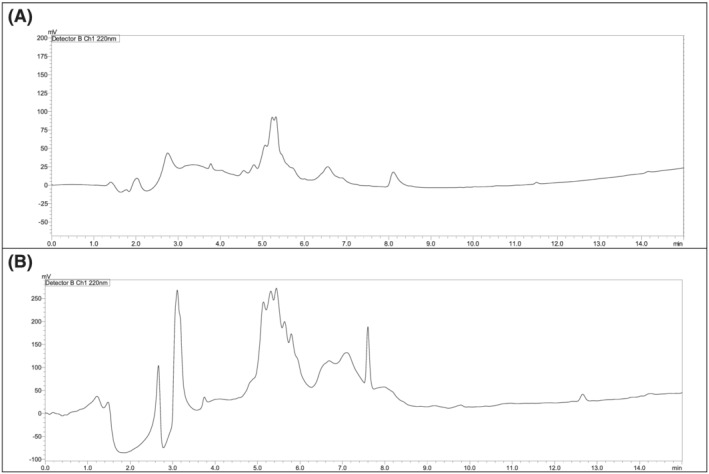
Chromatograms of TD2.2 BBBSp following the stepwise synthetic approach. (A) DIC/OxymaPure, (B) COMU/DIEA. 0%–50% of mobile phase B in 15 min. Mobile phase A (0.1% trifluoroacetic acid (TFA) in water), mobile phase B (0.1% TFA in ACN), flow rate 1 ml/min. Symmetry C_18_ column (150 × 3.9) mm, 5 μm

The Fmoc/*t*Bu strategy was considered using a combination of DIC and OxymaPure as coupling and additive reagents, respectively.^22^ The first attempt to synthesise the TD2.2 peptide was the common stepwise approach. However, this approach failed to deliver the target peptide after several attempts (Figure 2A). Another coupling paradigm was considered using COMU/DIEA but with no success either (Figure 2B).

By investigating the sequence composition of the BBBSp in this study, it comprises seven Arg amino acid residues, in addition to an appreciable number of hydrophobic amino acid residues. Moreover, in some regions of the sequence, these “difficult” amino acids are quite overcrowded. Hence, difficulties in the coupling and deprotection steps are expected to take place, which will fuel various side reactions that will compromise the final purity of the target peptide. Thus, as the chain gets longer, this phenomenon will go along with each step of the stepwise approach and eventually lead to the failure of the whole process.

While the stepwise approach failed to deliver the target BBBSp, we decided to follow the fragment condensation approach on a solid‐phase setting. Provided that having less coupling and deprotection steps between fragments helps in enhancing the yield and the purity of the final peptide with respect to the stepwise approach. Given that a strategic Gly is just located at the 15th position of the sequence, the peptide was divided into two fragments. Thus, the first 15 amino acids (SYWYRIVLSRTGRNG) were assembled on 2‐chlorotrityl chloride (CTC) resin to enable the cleavage of protected fragments to be later coupled with other fragments as appropriate.

**FIGURE 3:**
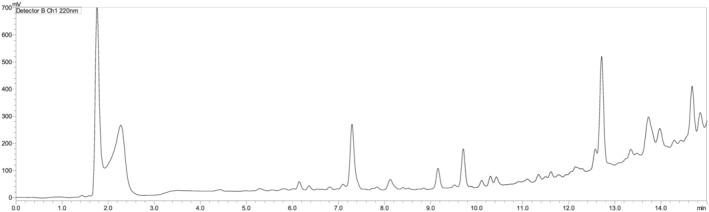
Chromatograms of Fmoc‐SYWYRIVLSRTGRNG‐OH protected fragments on CTC resin, *t*_R_ = 12.7 min; 5%–95% of mobile phase B in 15 min. For the rest of the chromatographic conditions, refer to the legend of Figure 2.

**FIGURE 4:**
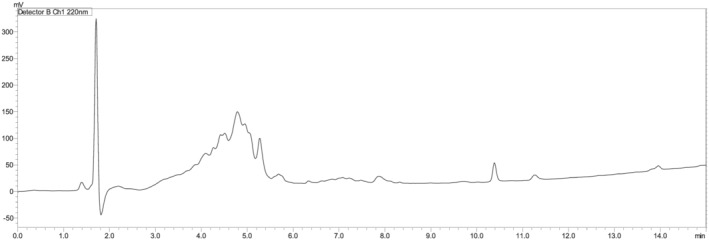
Chromatogram of the (H‐RLRVGRERPVLGESP‐NH_2_) fragments on Rink amide resin. For the chromatographic conditions, refer to the legend of Figure 3.

The second fragment also consisted of 15 amino acids (RLRVGRERPVLGESP), which were assembled on Rink amide resin to render an amide peptide, on which the first protected fragment will be condensed to obtain the final peptide. While the first fragment (Fmoc‐S(*t*Bu)Y(*t*Bu)W(Boc)Y(*t*Bu)R(Pbf)IVLS(*t*Bu)R(Pbf)T(*t*Bu)GR(Pbf)N(Trt)G‐OH) showed high yield (95.1%) and acceptable purity (74.1%) (Table 1 and Figure 3) with confirmation of its mass (Supporting Information Figure S1), the second fragment (H‐R(Pbf)LR(Pbf)VGR(Pbf)E(*t*Bu)R(Pbf)PVLGE(*t*Bu)S(*t*Bu)P‐NH_2_) showed poor purity; hence, it could not be used in further reaction steps. This urged us to shorten its sequence to smaller fragments (Figure 4).

**TABLE 1:** Analytical data of the peptide fragments and the final TD2.2

Peptide sequence	Yield %	Purity %	Mass	
			Calculated (*m/z*)	Mass found (*m/z*)
Fmoc‐S(*t*Bu)Y(*t*Bu)W(Boc)Y(*t*Bu)R(Pbf)IVLS(*t*Bu)R(Pbf)T(*t*Bu)GR(Pbf)N(Trt)G‐OH)	92.9	74.1	3430.28	1146.87 [M+3H]^3+^
Fmoc‐R(Pbf)LR(Pbf)VG‐OH	98.3	96.3	1326.64	1327.04 [M+H]^+^
				664.38 [M+2H]^2+^
Fmoc‐R(Pbf)E(*t*Bu)R(Pbf)PV‐OH	99.1	88.9	1438.76	1440.36 [M+H]^+^
				721.8 [M+2H]^2+^
H‐LGESP‐NH_2_	97.1	100.0	500.55	501.57 [M+H]^+^

The difficulties in the second fragment are ascribed to the presence of six hydrophobic residues which are thought to hinder the coupling and Fmoc removal steps. Furthermore, this fragment also encompasses four Arg residues in a very close proximity to each other. Moreover, these Arg residues were localised over a short part of this fragment and each pair was separated by only one amino acid. Thus, this position was making the coupling reaction more demanding as a result of the steric hindrance inferred by the large size of Fmoc‐Arg(Pbf)‐OH. On the other hand, by looking at the sequence of the first fragment, there are less hydrophobic residues (four residues) and their presence is being mitigated by the presence of more polar and hydrophilic residues. Also, there are less Arg residues (three residues), which are not congested over a short part of the sequence but rather are spread out over the entire 15‐aa sequence with an appreciable distance between them. This might be a plausible explanation for the difficulties experienced in the synthesis of the second fragment with respect to the first one.

**FIGURE 5:**
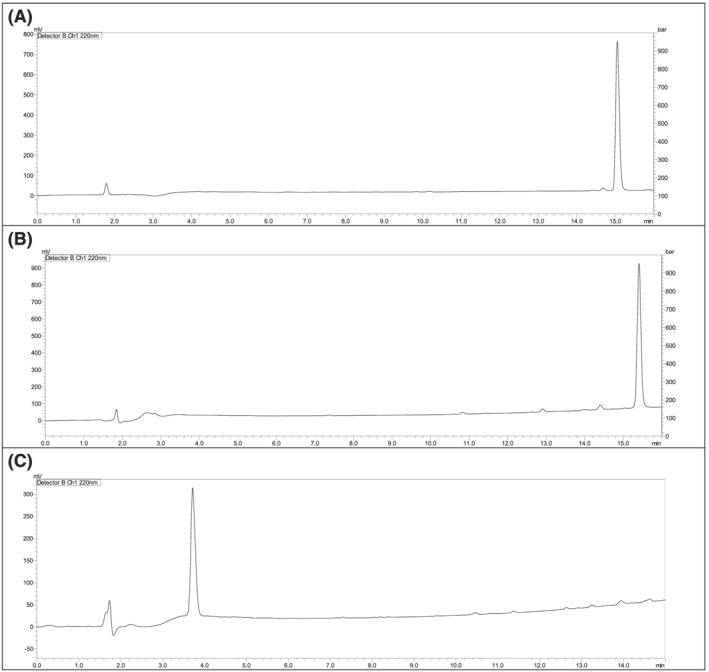
Chromatograms of (A) Fmoc‐RLRVG‐OH, *t*_R_ = 15.0 min; (B) Fmoc‐RERPV‐OH, *t*_R_ = 15.4 min; (C) H‐LGESP‐NH_2_ unprotected fragment, *t*_R_ = 3.7 min. For the chromatographic conditions, refer to the legend of Figure 3.

A decision was made to shorten the (H‐RLRVGRERPVLGESP‐NH_2_) fragment into two peptide fragments comprising 10 and five residues. While the 5‐mer fragment was assembled on CTC resin, the other 10‐mer fragment was assembled on Rink amide resin. The 5‐mer peptide fragment (Fmoc‐R(Pbf)LR(Pbf)VG‐OH) showed high yield (98.3%) and purity (96.3%) (Table 1 and Figure 5A).

**FIGURE 6:**
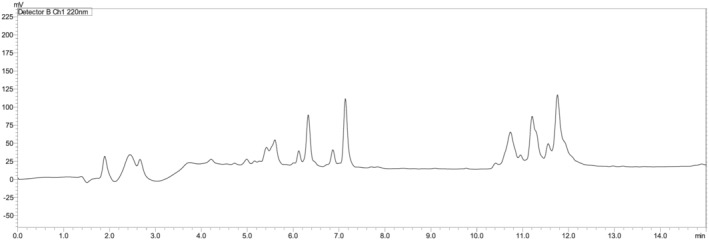
Chromatogram of the (H‐RERPVLGESP‐NH_2_) decapeptide fragment on Rink amide resin. For the chromatographic conditions, refer to the legend of Figure 3.

On the contrary, the purity of the H‐RERPVLGESP‐NH_2_ decapeptide was still poor and unsuitable to be used in subsequent reactions (Figure 6).

Lastly, dividing the second 15‐amino acids fragment into three fragments of five amino acids each (Fmoc‐R(Pbf)LR(Pbf)VG‐OH, Fmoc‐R(Pbf)E(*t*Bu)R(Pbf)PV‐OH, and H‐LGESP‐NH_2_) gave the best yields and purities for all of them (98.3, 96.3)%, (99.1, 88.9)%, and (97.1, 100.0)%, respectively (Table 1 and Figure 5A–C) and their masses were confirmed (Supporting Information Figures S2–S4). It is worth highlighting that in the first attempt of synthesising the Fmoc‐R (Pbf)LR(Pbf)VG‐OH fragment, a significant peak preceding the main peak was observed (40.7%) (Supporting Information Figure S5), where the observed mass of 1213 [M+H]^+^ confirms it is the ‘des‐Leu’ analogue (Figure 5A). However, the synthesis was repeated, and this deletion peptide was significantly reduced (1.4%) by double coupling the Arg and the subsequent residue (Leu). Hence, we recommend this practice for a purer product. Of note, it is worth mentioning that all the synthesised fragments were freely soluble in DMF.

**FIGURE 7:**
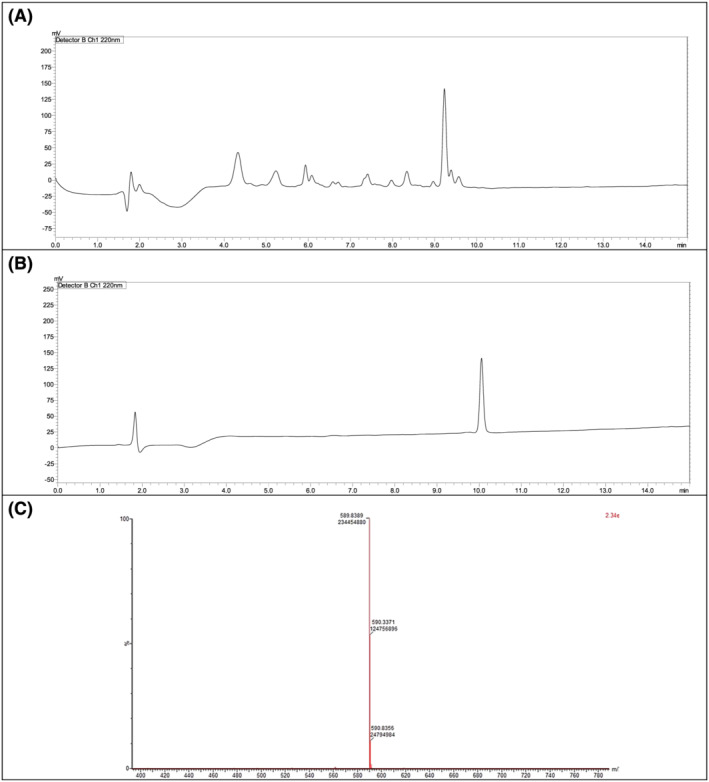
(A) Chromatogram of the TD2.2 peptide following the fragment condensation synthetic approach [15 + 5 + 5 + 5], *t*_R_ = 9.2 min. (B) Chromatogram of the purified TD2.2 BBBSp. (C) LCMS chromatogram. For the chromatographic conditions, refer to the legend of Figure 2.

The four fragments were subsequently assembled with an overall purity of 71%. Table 1 shows the confirmation of the mass for the four peptide fragments. The peptide was then easily purified, and its mass was confirmed as well (Figure 7).

**FIGURE 8:**
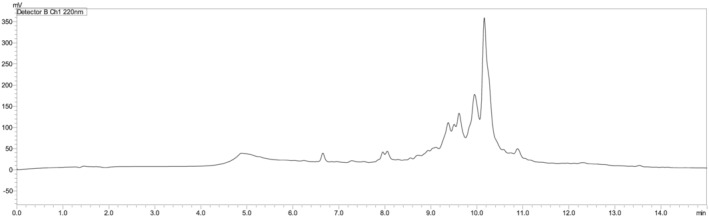
Chromatogram of the TD2.2 peptide using the LibertyBlue microwave‐assisted automatic synthesiser. For the chromatographic conditions, refer to the legend of Figure 2.

While the obtained peptide was obtained with about 71% purity, it was also synthesised using the microwave‐assisted automatic synthesiser and a purity of around 79% was achieved (Figure 8).

Thus, in this work, we were able to manually synthesise a TD2.2 BBBSp following the fragment condensation approach on solid‐phase mode with a purity close to the one achieved by the automatic synthesiser. However, as mentioned earlier, we consider the current study friendlier than the automatic peptide synthesiser as it requires less chemicals and energy. For instance, to synthesise 0.05 mmol scale of a 30‐mer peptide, the synthesiser consumes about 1000 ml DMF, 120 ml deprotection solution, 60 ml DIC and 30 ml OxymaPure, while in the manual approach, these quantities are dramatically reduced as follows: 200 ml DMF, 30 ml deprotection solution, 5 ml DIC and 5 ml OxymaPure. In addition, greening the process would be easier in the manual synthesis in terms of using different reagents and the needed optimisation steps afterwards.

It is worth mentioning that the fragments were directly coupled without prior purification to prove the efficiency of this approach versus the stepwise one. However, the fragments could be purified prior to the coupling process as needed.

## 4 CONCLUSIONS

Successful synthesis of TD2.2 BBBSp was demonstrated in this work. While the stepwise SPPS failed to deliver the target peptide, the convergent approach was able to deliver the peptide with a comparable purity to that obtained by the microwave‐assisted automatic synthesiser. Choosing the correct fragment with the correct amino acid composition is key in fragment condensation work to avoid shortcomings that could drive the whole synthetic process to failure.

We consider this approach to be friendlier to the environment as less reagents were required than those needed with the automated approach. This would translate in cutting the cost of the whole process should the manual approach be considered. It is worth noting that the automatic peptide synthesiser is a high throughput tool with reduced coupling times. However, it consumes fivefold more of DMF, deprotection solution, coupling agents and additives.

After the promising work conducted and explained in this paper, we are now exploring new synthetic methodologies such as the one‐bead one‐compound (OBOC) approach^34^ to prepare new families of peptides. This technique will be employed to synthesise this BBBSp as well. In our lab, we are also developing a new high‐throughput method based on chromatography that will assist in the selection of the peptide sequences for glial cell targeting.

## CONFLICT OF INTEREST

None to declare.

## Supporting information


**Figure S1.** Mass of Fmoc‐S(*t*Bu)Y(*t*Bu)W(Boc)Y(*t*Bu)R(Pbf)IVLS(*t*Bu)R(Pbf)T(*t*Bu)GR(Pbf)N(Trt)G‐OH)
**Figure S2.** Mass of H‐LGESP‐NH_2_
**Figure S3.** Mass of Fmoc‐R(Pbf)E(*t*Bu)R(Pbf)PV‐OH
**Figure S4.** Mass of Fmoc‐R(Pbf)LR(Pbf)VG‐OH
**Figure S5.** Chromatograms of Fmoc‐RLRVG‐OH, t_R_ = 14.8 min, and the des‐Leu analogue, t_R_ = 14.2 min. For chromatographic conditions, refer to the legend of Figure 2.
