# Supplementary material for: Successful synthesis of a glial‐specific blood–brain barrier shuttle peptide following a fragment condensation approach on a solid‐phase resin
Source: J Pept Sci. 2022 Aug 28;29(2):e3448. doi: 10.1002/psc.3448 (PMC10078400; doi:10.1002/psc.3448)
Supplement: Supplementary file 1 — Figure S1. Mass of Fmoc‐S(tBu)Y(tBu)W(Boc)Y(tBu)R(Pbf)IVLS(tBu)R(Pbf)T(tBu)GR(Pbf)N(Trt)G‐OH) Figure S2. Mass of H‐LGESP‐NH2 Figure S3. Mass of Fmoc‐R(Pbf)E(tBu)R(Pbf)PV‐OH Figure S4. Mass of Fmoc‐R(Pbf)LR(Pbf)VG‐OH Figure S5. Chromatograms of Fmoc‐RLRVG‐OH, tR = 14.8 min, and the des‐Leu analogue, tR = 14.2 min. For chromatographic conditions, refer to the legend of Figure 2. [file PSC-29-0-s001.docx]

**Successful synthesis of a glial-specific blood-brain barrier shuttle peptide following a fragment condensation approach on a solid-phase resin**

**Othman Al Musaimi^1, 2*,^ Sophie V. Morse^3*^, Lucia Lombardi^1, 2^, Simona Serban^4^, Alessandra Basso^4^, and Daryl R. Williams^1^**

1. Department of Chemical Engineering, Imperial College London, London, UK
2. The Sargent Centre for Process Systems Engineering, Imperial College London, London, UK
3. Department of Bioengineering, Imperial College London, London, UK
4. Purolite, Unit D Llantrisant Business Park, Llantrisant, UK

* Correspondance: (O. A. M.) [o.al-musaimi@imperial.ac.uk](mailto:o.al-musaimi@imperial.ac.uk); (S. V. M.) sophie.morse11@imperial.ac.uk


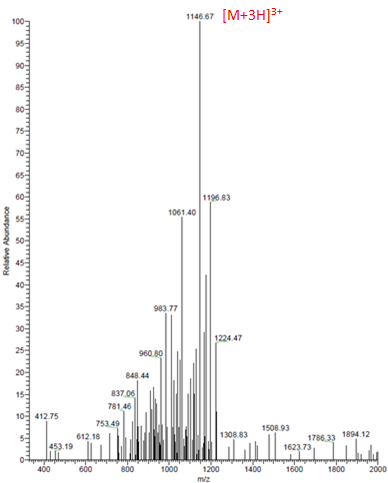


Supplementary Figure 1. Mass of Fmoc-S(*t*Bu)Y(*t*Bu)W(Boc)Y(*t*Bu)R(Pbf)IVLS(*t*Bu)R(Pbf)T(*t*Bu)GR(Pbf)N(Trt)G-OH)


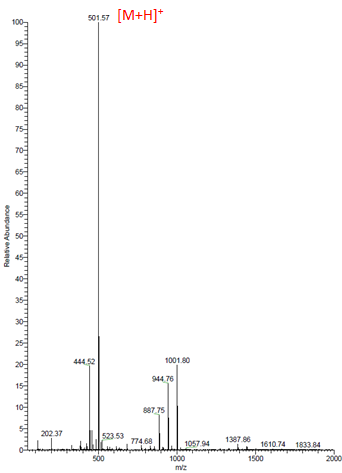


Supplementary Figure 2. Mass of H-LGESP-NH_2_


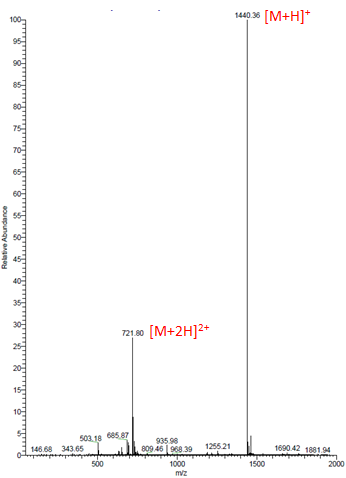


Supplementary Figure 3. Mass of Fmoc-R(Pbf)E(*t*Bu)R(Pbf)PV-OH


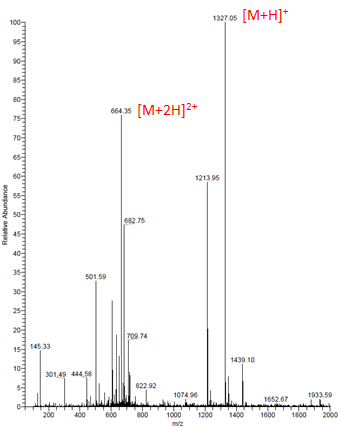


Supplementary Figure 4. Mass of Fmoc-R(Pbf)LR(Pbf)VG-OH

Supplementary Figure 5. Chromatograms of Fmoc-RLRVG-OH, t_R_= 14.8 min, and the des-Leu analogue, t_R_= 14.2 min. For chromatographic conditions, refer to the legend of Figure 2.
